# Evaluating the individuality of animal‐habitat relationships

**DOI:** 10.1002/ece3.4554

**Published:** 2018-10-03

**Authors:** Robert A. Montgomery, Kyle M. Redilla, Waldemar Ortiz‐Calo, Trenton Smith, Barbara Keller, Joshua J. Millspaugh

**Affiliations:** ^1^ The Research on the Ecology of Carnivores and their Prey Laboratory Department of Fisheries and Wildlife Michigan State University East Lansing Michigan; ^2^ School of Natural Resources University of Missouri Columbia Missouri; ^3^ Missouri Department of Conservation Columbia Missouri; ^4^ Wildlife Biology Program, W.A. Franke College of Forestry and Conservation University of Montana Missoula Montana

**Keywords:** Bayesian, *Cervus elaphus*, ecological inference, elk, resource selection function, telemetry

## Abstract

Examining the ways in which animals use habitat and select resources to satisfy their life history requirements has important implications for ecology, evolution, and conservation. The advent of radio‐tracking in the mid‐20th century greatly expanded the scope of animal‐habitat modeling. Thereafter, it became common practice to aggregate telemetry data collected on a number of tagged individuals and fit one model describing resource selection at the population level. This convention, however, runs the risk of masking important individuality in the nature of associations between animals and their environment. Here, we investigated the importance of individual variation in animal‐habitat relationships via the study of a highly gregarious species. We modeled elk (*Cervus elaphus*) location data, collected from Global Positioning System (GPS) collars, using Bayesian discrete choice resource selection function (RSF) models. Using a high‐performance computing cluster, we batch‐processed these models at the level of each individual elk (*n* = 88) and evaluated the output with respect to: (a) the composition of parameters in the most supported models, (b) the estimates of the parameters featured in the global models, and (c) spatial maps of the predicted relative probabilities of use. We detected considerable individual variation across all three metrics. For instance, the most supported models varied with respect to parameter composition with a range of seven to 17 and an average of 14.4 parameters per individual elk. The estimates of the parameters featured in the global models also varied greatly across individual elk with little conformity detected across age or sex classes. Finally, spatial mapping illustrated stark differences in the predicted relative probabilities of use across individual elk. Our analysis identifies that animal‐habitat relationships, even among the most gregarious of species, can be highly variable. We discuss the implications of our results for ecology and present some guiding principles for the development of RSF models at the individual‐animal level.

## 1 INTRODUCTION

Quantifying animal‐habitat relationships is a cornerstone of ecological inquiry providing insights across both theoretical and applied dimensions (Johnson, 1980; Morris, 2003). Research on this topic has implications for optimal foraging, predator–prey interactions, survivorship, reproduction, life history, and, correspondingly, population‐level processes (Charnov, 1976; MacArthur & Pianka, 1966; Morris, 2003). While a variety of data collection methods are used to document animal‐habitat relationships, telemetry (both Global Positioning System—GPS and Very High Frequency) tends to be among the most widely used techniques (Kenward, 2001; Montgomery & Roloff, 2013; Thomas & Taylor, 2006). Telemetry technology has expanded dramatically in the last 50 years (Hebblewhite & Haydon, 2010) with coupled growth in the quantitative methods used to model resultant data (Avgar, Potts, Lewis, & Boyce, 2016; Gaillard et al., 2010; Hirzel & Le Lay, 2008). Formative techniques for quantifying animal‐habitat relationships include, but are not limited to, compositional analysis (Aebischer, Robertson, & Kenward, 1993), logistic regression (Thomasma, Drummer, & Peterson, 1991), discrete choice analysis (Cooper & Millspaugh, 1999), maximum entropy (Phillips, Anderson, & Schapire, 2006), ecological niche factor analysis (Hirzel, Hausser, Chessel, & Perrin, 2002), random forests (Cutler et al., 2007), and point process models (Renner et al., 2015).

Most of these models operate in a similar fashion via the comparison of habitat units (clusters of resources) that are used (i.e., where animal occurrence has been documented) to those that are considered to be either unused or available (i.e., where animal occurrence *did* or *may* not have occurred). The statistical comparison of used to unused/available habitat units was unified under the broad framework of the resource selection function (RSF; Boyce & McDonald, 1999; Manly, McDonald, Thomas, McDonald, & Erickson, 2002). In this way, RSF models estimate the relative probability that a habitat unit is used, given the resources present, in relation to available or unused habitat units (Manly et al., 2002). While an RSF is assumed to be proportional to a resource selection probability function (RSPF) up to an arbitrary constant, this proportionality is not guaranteed (Keating & Cherry, 2004; Rota et al., 2013; Royle, Chandler, Yackulic, & Nichols, 2012). Thus, given reasons of convenience, precedence, and evident misconceptions regarding RSPF parameter estimation, RSF models remain a widely used framework for assessing animal‐habitat relationships (Manly et al., 2002; Rota et al., 2013; Thomas & Taylor, 1990, 2006). While there are important sampling elements to consider when devising an RSF analysis including, and arguably most importantly, the means by which available or unused habitat units are measured, the ecological inferences garnered from RSF models tend to be applied, in that they are typically devised to inform prevailing conservation and management practice (Aarts, MacKenzie, McConnell, Fedak, & Matthiopolous, 2008; Keating & Cherry, 2004; Montgomery & Roloff, 2013). Given that conservation and management actions are most often applied at the population level (Johnson and Gillingham, 2008; Hooten, Buderman, Brost, Hanks, & Ivan, 2016), it has become common practice to deploy telemetry collars/tags on a number of animal subjects and then aggregate the resultant locational data fitting one RSF model (Thomas & Taylor, 2006).

The process of aggregating telemetry data across animal subjects, however, can obscure individual variation in animal‐habitat relationships, potentially biasing inference (Benson, Sikich, & Riley, 2016; Marzluff, Millspaugh, Hurvitz, & Handcock, 2004; Millspaugh et al., 2006). This bias may be particularly evident when the number of telemetry locations is unbalanced (given disparities in data collection effort, duration of study, or technological problems associated with the telemetry technology) across individual animals under study (Aarts et al., 2008; Gillies et al., 2006). Thus, in an effort to account for individual variation, RSF models are sometimes fit with a random effect representing animal ID (Gillies et al., 2006). This random effect (which can be fit both as a random intercept or a random slope) relaxes the independence assumption by allowing the parameter estimates to vary according to a population‐level probability distribution (Aarts et al., 2008; Duchesne, Fortin, & Courbin, 2010; Gillies et al., 2006; Hebblewhite & Merrill, 2008; Hooten et al., 2016; Mysterud & Ims, 1998; Thomas, Johnson, & Griffith, 2006). Nevertheless, recent research has demonstrated that individual animal behavior can be importantly variable, even among social species (Bartelt & Klaver, 2017; Dall, Bell, Bolnick, & Ratnieks, 2012; Frost, Winrow‐Giffen, Ashley, & Sneddon, 2007; Réale, Dingemanse, Kazem, & Wright, 2010; Spiegel, Leu, Bull, & Sih, 2017). Such variation raises questions about the extent to which individuality should be considered among animal‐habitat relationship models (Merrick & Koprowski, 2017; Pape & Löffler, 2015). The risk is that by treating all individuals similarly and aggregating locational data post hoc, the potential to misidentify resource selection, confounding inference and complicating prevailing management or conservation practice, increases. Therefore, studies examining individuality in animal‐habitat relationships are needed (Marzluff et al., 2004; Thomas et al., 2006).

Here, we investigated individual variation in animal‐habitat relationships for a highly gregarious species. We studied elk (*Cervus elaphus*) resource selection in southern Missouri, USA. Using a high‐performance computing (HPC) cluster, we fit Bayesian discrete choice RSF models to each individual elk in the study (*n* = 88) and compared the output of these models across three areas of inference common in RSF analyses: (a) the composition of parameters in the most supported RSF models, (b) the estimated RSF parameters from the global models, and (c) maps depicting the predicted relative probabilities of use. Additionally, we evaluated these metrics among a model fit to all individual elk to provide a comparison between individual‐level modeling and the aggregate population‐level approach, which is currently the convention in animal‐habitat relationship research. We discuss the implications of this study for ecological inference and provide guidance on the ways in which RSF models can be efficiently developed at the individual‐animal level.

## 2 MATERIALS AND METHODS

### 2.1 Study area

We radio‐tracked elk (*n* = 88) in the Peck Ranch Conservation Area, a 9,327 ha plot of land managed by the Missouri Department of Conservation (MDC), in the Missouri Ozarks. These elk were part of a large restoration effort to the elk restoration zone of Missouri (MDC, 2010). All capture and handling protocols were approved by the University of Missouri Animal Care and Use Committee (Protocol 6909). We fit all elk ≥1 year‐old with a GPS telemetry collar (RASSL custom 3D cell collar; North Star Science and Technology, LLC, King George, VA, or G2110E Iridium/GPS series model; Advanced Telemetry Systems, Isanti, MN). All collars, apart from two (2.2%) which were set to 2‐hr fix attempt schedules, were programed to record a GPS location every 5 hrs.

Using the locational data returned from these telemetry systems, we measured environmental variables present at the used and available habitat units. Available habitat units were delimited using the radius of available habitat method (Durner et al., 2009). We established a buffer around each used habitat unit with a radius equal to *c* (*a* + 2*b*), where *a*,* b*, and *c* represent the mean hourly movement rate, the standard deviation of the movement rate, and the number of hours between visits to habitat units, respectively. We then randomly sampled five habitat units within these buffers to determine the available habitat units for the corresponding used habitat units, hereafter referred to as a “choice set.”

### 2.2 Environmental variables

We developed a geographic information database including 11 environmental variables, each depicted as rasters at a resolution of 30 m. This database consisted of percent tree canopy cover (2011 US Forest Service National Land Cover Database, http://www.mrlc.gov/nlcd11_data.php, accessed 8 January 2015), number of years since prescribed burn, aspect (degrees), slope (percent), distance to wooded edge (m), the interspersion and juxtaposition index (Griffith, Martinko, & Price, 2000), road density (km paved or gravel road/km^2^ within 95 km^2^ circle), distance to paved road (m), distance to closed two‐track roads (m), and distance to public gravel roads (m). For more information on the development of these rasters, see Smith (2015). Finally, we considered habitat type as a categorical covariate. This variable had eight categories including warm‐season grassland, cool‐season grassland, shrubland, savannah (20%–50% canopy cover), woodland (50%–80% canopy cover), forest (>80% canopy cover), glade, and forage opening.

### 2.3 RSF modeling

We used Bayesian discrete choice RSF models to describe elk‐habitat relationships because they can define *availability* separately for each used habitat unit, providing a more realistic depiction of resource selection (Cooper & Millspaugh, 1999, 2001; McCracken, Manly, & Vander Heyden, 1998). A Bayesian framework was most appropriate given the flexibility to accommodate random effects (Browne & Draper, 2006; Gelman et al., 2013). This model is defined as follows, where the probability of an individual elk choosing alternative *l* from a set of *C* feasible habitat units at unit *i* is given by

(1) $$P_{\mathrm{il}}=\frac{e^{\beta X_{\mathrm{il}}}}{\sum_{c=1}^{C}e^{\beta X_{\mathrm{ic}}}},$$

where

(2) $$\beta X_{\mathrm{il}}=\beta_{1}X_{il1}+\beta_{2}X_{il2}+\ldots +\beta_{k}X_{\mathrm{ilk}},$$

is the *utility* of unit *l* to the individual being considered, consisting of *k* slope parameters measured on each used and available unit.

We developed this model in the Bayesian package Stan (Stan Development Team 2016a) using R and RStan as an interface (R Core Team 2016; Stan Development Team 2016b). For each individual‐elk model, we used four chains of 1,000 draws each, with a burn‐in period of 200. In most cases, 1,000 iterations were more than satisfactory to reach convergence to the posterior using Stan, so replicating this across four chains provided a reasonable baseline for estimating the individual models (Vehtari, Gelman, & Gabry, 2017). Given computational challenges, we used a HPC cluster to fit all individual‐level models remotely and in parallel (Institute for Cyber‐Enabled Research, Michigan State University). We assessed convergence of all models by ensuring that the potential scale reduction factor, $\hat{R}$, for all parameters was <1.1, and the effective sample size, ${\hat{n}}_{\mathrm{eff}}$, was >100 (Gelman et al., 2013). We calculated goodness of model fits using posterior predictive checks, whereby we computed the probability that a test statistic, *T*, calculated on new data simulated from our model, y*, is more extreme than *T* calculated on our observed data, *y* (Gelman et al., 2013; Hobbs & Hooten, 2015). We used the chi‐square test statistic to conduct these checks for the all individual‐level global models as follows;

(3) $$\begin{array}{c} \text{Pr}(T\left(y^{*},\theta \right)\geq T\left(y,\theta \right)|y)\\ T\left(y,\theta \right)=\sum_{i}\frac{{\left(y_{i}-p_{i}\right)}^{2}}{p_{i}}, \end{array}$$

where *θ* represents the parameters of the fitted model, and *p*_*i*_ is the probability associated with the *i*th choice. The first expression in (3) returns a Bayesian *p*‐value, *p*_B_, which we used to diagnose lack of model fit (Gelman et al., 2013; Hobbs & Hooten, 2015).

We then assessed patterns of individual variation in resource selection by comparing: (a) the number and composition of parameters included in the most supported models, (b) the parameter estimates within the global models, and (c) the spatial maps of the predicted relative probability of use. To make comparisons based on the model selection approach, we fit all possible combinations of parameters, following our examination of collinearity, for each individual elk (Cade, 2015; Wiens, Dale, Boyce, & Kershaw, 2008). Before model fitting, we examined evident collinearity among the environmental variables and excluded redundant environmental variables until all pairwise correlations were |*r*| ≤ 0.6. We ranked models using Watanabe‐Akaike information criterion (WAIC; Watanabe, 2010), providing a Bayesian and computationally efficient model selection tool (Vehtari, Gelman, & Gabry, 2015), where the most supported model was the one with the lowest WAIC score (Gelman, Hwang, & Vehtari, 2014; Vehtari et al., 2015; Watanabe, 2010). We then calculated “inclusion rates” of the parameters featured in the top 5% of models for each individual elk. Finally, we produced spatial maps of the predicted relative probabilities of use for the most supported models of all individual elk expressed across the entire elk restoration zone.

### 2.4 Comparison with population‐level RSF

To provide a comparison with conventional animal‐habitat relationship research, we extended Equations (1) and (2) to develop a model fit to the resultant telemetry data for all individual elk aggregated together. Here, we made explicit the probability of individual *j* choosing alternative *l* from a set of *C* feasible alternatives to unit *i*, defined as

(4) $$P_{\mathrm{ijl}}=\frac{e^{\beta_{j}X_{\mathrm{ijl}}}}{\sum_{c=1}^{C}e^{\beta_{j}X_{\mathrm{ijc}}}},$$

with utility function now defined as

(5) $$\begin{array}{c} \beta_{j}X_{\mathrm{ijl}}=\beta_{j1}X_{ijl1}+\beta_{j2}X_{ijl2}+\ldots +\beta_{\mathrm{jk}}X_{\mathrm{ijlk}}\\ \text{and}\;\beta_{\mathrm{jk}}∼Normal\left(\mu_{k},\sigma_{k}^{2}\right), \end{array}$$

where the individual parameters for selection of environmental variable *k* (the *β*_*jk*_ for all *j *=* *1, 2, …, 88 individuals), are assumed to be normally distributed random effects following some population distribution. The parameters of the population distributions for each environmental variable *k* (*μ*_*k*_ and $\sigma_{k}^{2}$) are referred to as hyperparameters (Hobbs & Hooten, 2015). Thus, we fit a hierarchical random slopes model, where inference can be made on the central tendency of selection for each environmental variable *k*, or the mean hyperparameter *μ*_*k*_, as well as variation among individuals, or the standard deviation hyperparameter *σ*_*k*_. We conducted our population‐level model estimation using the following uninformative priors for all hyperparameters:

(6) $$\begin{array}{c} \mu_{k}∼Normal\left(0,10\right)\\ \sigma_{k}∼Uniform\left(0,10\right). \end{array}$$

As above with the individual‐level models, we developed the same three metrics of evaluation (i.e., composition of parameters in the most supported model, estimates of the parameters in the global model, and spatial maps of the predicted relative probability of use). We compared the spatial maps between the individual and population level by computing a Spearman rank correlation coefficient, *ρ*.

## 3 RESULTS

We radio‐tracked 88 elk between June 1, 2011 and September 15, 2014 (35 elk released in 2011 cohort [22 female, 13 male], 24 elk released in 2012 cohort [17 female, 7 male], and 29 elk released in 2013 cohort [26 female, 3 male]). Individual‐level RSF models were estimated based on 95 to 4,865 choice sets, depending on the elk, with >50% of the models comprised of between 783 and 2,071 choice sets. We detected high collinearity (0.61 ≤ |*r*| ≤ 0.84.) between the distance to gravel road and road density variables for all individuals, and we excluded the former variable from consideration given that two other variables (distance to paved road and distance to two‐track road) quantified proximity to roads. All individual models achieved convergence ($\hat{R}$ < 1.1) and Bayesian *p*‐values averaged 0.33 (range 0.16–0.58) indicating good model fit. At the population level, we fit our global RSF model and sub‐models using 141,197 choice sets. The Bayesian *p*‐value test for the global population‐level model indicated no lack of fit (*p*_B_ = 0.35).

### 3.1 Composition of parameters

**Figure 1:**
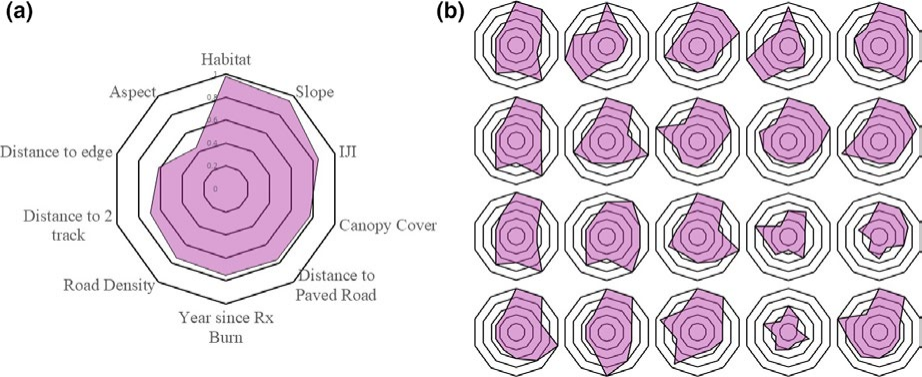
Radial plots which display the average number of parameters among the most supported (as ranked by Watanabe‐Akaike information criterion [WAIC]) resource selection function (RSF) models among all elk (a) and the most supported RSF models for 20 randomly selected individual elk (b) reintroduced into the Missouri Ozarks (2011–2014)

Parameter composition among the top 5% of individual elk models varied considerably as evidenced by radial plots of 20 randomly selected elk (Figure 1). For example, aspect was only included as a predictor in the most supported model for 44% (39 of the 88 most supported models) of the individual elk. Habitat and slope, however, were included in the most supported models for virtually all individual elk (Figure 1). The most supported individual‐level models had a range of seven to 17 parameters with an average of 14.4 parameters across all 88 individual elk. At the population level, the most supported model was the global model including at 17 parameters.

### 3.2 Parameter estimates

**Figure 2:**
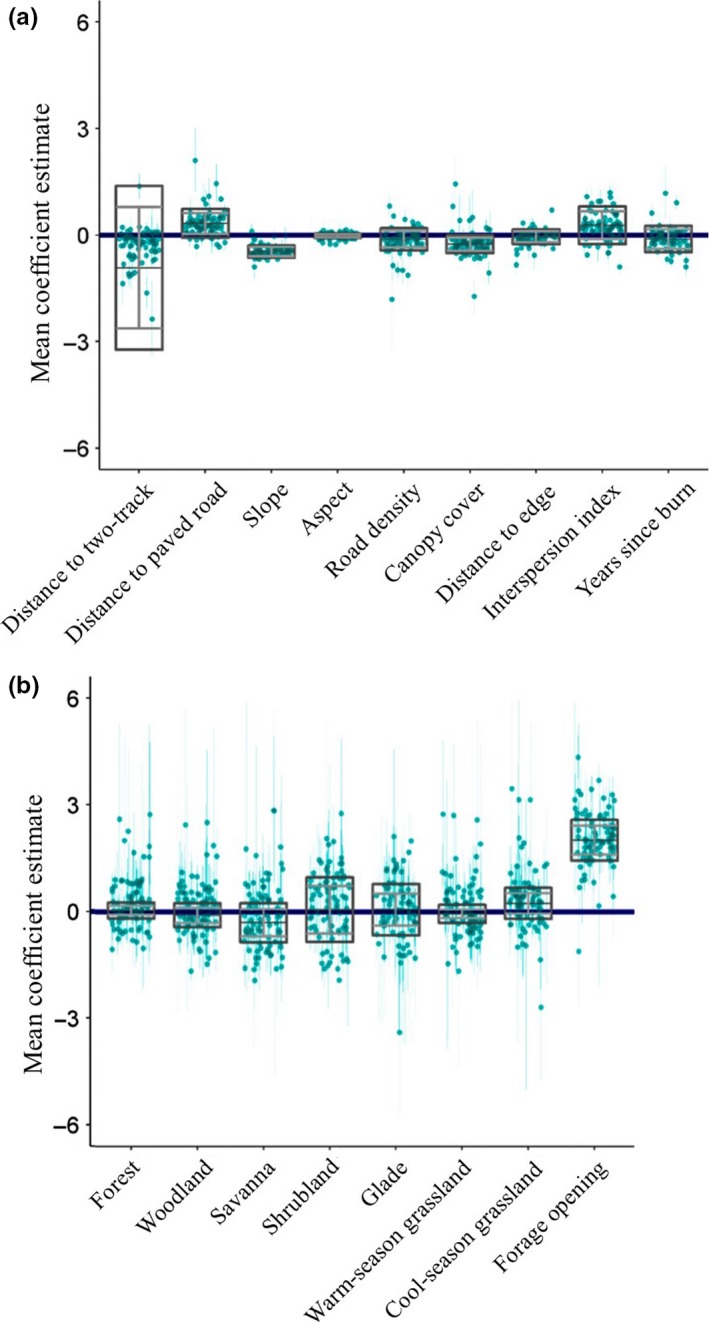
The individual‐level RSF parameter estimates (green dots) with 95% Bayesian credible intervals (CIs) for reintroduced elk in the Missouri Ozarks (2011–2014). The total number of parameters are divided between panels (a) and (b). Also included are the population‐level random effects distributions (boxes). The middle horizontal bars within the boxes are the point estimates of the mean hyperparameters of the random effects distributions, the gray horizontal bars are the standard deviation of the hyperparameters, and the ends of the boxes represent 95% Bayesian credible intervals on the mean + *SD* point estimates

Estimates of the parameters within the global models of individual elk also varied (Figure 2). Uncertainty around those estimates was high for most individual elk, as evidenced by the large 95% Bayesian credible intervals (CIs) around these estimates (Figure 2). The population‐level random effects distributions are depicted by the boxes (Figure 2) where the middle line of each box represents the point estimates of the mean hyperparameters, the horizontal bars are the standard deviation of the hyperparameters, and the ends of the boxes are the 95% Bayesian CIs on the mean plus the standard deviation point estimates. At the population level, 13 of the 17 *μ*_*k*_ estimates had 95% CIs which did not overlap zero. These included estimates for all *μ*_*k*_ of the continuous environmental variables as well as all categorical habitat types except forest, shrubland, glade, and warm‐season grassland. However, the estimated 95% random‐effect CIs did not contain zero for two variables including slope (Figure 2a) and forage opening (Figure 2b). Population‐level point estimates of individual variation (the *σ*_*k*_ estimates) ranged from a minimum of 0.04 for aspect to 1.71 for distance to two tracks. The latter was large relative to estimates of *σ*_*k*_ for the remaining environmental variables, as all other estimates fell below 0.67 (Figure 2).

### 3.3 Predicted relative probabilities of use

**Figure 3:**
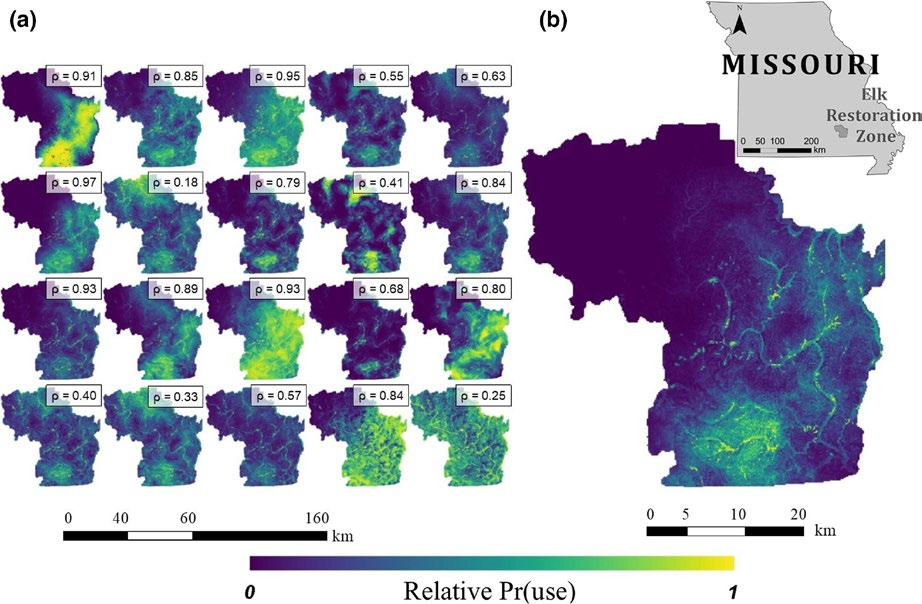
The spatial maps of the predicted relative probabilities of use for 20 randomly selected individual elk (a) reintroduced to the Missouri Ozarks (2011–2014) compared to the population‐level RSF (b). Spearman rank correlations (*ρ*; see Table 1) between each individual‐level prediction and the population‐level prediction are also shown

Spatial maps of the predicted relative probability of use revealed differing patterns of resource selection among individual elk across the elk restoration zone of Missouri (Figure 3). As a means of visually assessing the variation in these predictions, we randomly selected an array of 20 predictive maps (Figure 3a). These maps exhibited considerable variation in the predicted relative probabilities of use among individual elk (Figure 3a). This variation was also apparent when comparing the individual‐level maps to the population‐level map (Figure 3b). This variation was particularly apparent in the northwest and southeast portions of the study area, but differences were also apparent in the degree to which hotspots of high relative probability of use were spatially clustered (Figure 3). The predictive maps of 44% (39 of 88) of the individual elk were uncorrelated (*ρ* ≤ 0.6) with the population‐level prediction (Table 1). An additional 6% (5 of 88) of the individual elk predictive maps were negatively correlated with the population‐level predictive map (Table 1). Finally, none of the three metrics assessed exhibited any obvious conformity in patterning based on individual elk age, sex, nor the cohort year in which it was fit with a GPS collar.

**Table 1:** Spearman's rank correlation (*ρ*) of pairwise comparisons between spatial maps of the predicted relative probability of use at the individual level and population level by elk reintroduced into the Missouri Ozarks (2011–2014; see Figure 3)

*ρ*	*N*	Proportion
*ρ* ≥ 0.9	12	0.14
0.8 ≤ *ρ* < 0.9	18	0.20
0.7 ≤ *ρ* < 0.8	10	0.11
0.6 ≤ *ρ* < 0.7	9	0.10
0.5 ≤ *ρ* < 0.6	8	0.09
0.4 ≤ *ρ* < 0.5	9	0.10
0.3 ≤ *ρ* < 0.4	6	0.07
0.2 ≤ *ρ* < 0.3	2	0.02
0.1 ≤ *ρ* < 0.2	8	0.09
0 ≤ *ρ* < 0.1	1	0.01
Negative *ρ*	5	0.06

## 4 DISCUSSION

Emergent technologies continue to push the field of animal‐habitat modeling forward. With advancements in remote sensing and animal tracking, we now have the ability to model animal‐habitat relationships with unprecedented resolution (Kays, Crofoot, Jetz, & Wikelski, 2015). However, methodological legacies, such as the practice of aggregating telemetry data prior to model fitting (Thomas & Taylor, 2006), can affect the ecological inferences drawn from these efforts. With an interest to better understand how variation in the decisions of individual animals can scale up to have population‐level consequences (Gaillard et al., 2010; Hebblewhite & Haydon, 2010), we examined the resource selection of elk in the Missouri Ozarks. We found that the composition of parameters in the most supported models, the estimates of parameters in the global models, and the predicted relative probabilities of use considerably varied by individual. In actuality, we could not detect any real conformity in elk‐habitat relationships by age, sex class, or the cohort year in which the animal was collared.

While the scope of our inferences is restricted to this population of elk in Missouri, we find the extent of the variation that we observed to be applicable to other systems and other species. As elk are highly gregarious and often expected to respond similarly to the environment (Haydon et al., 2008; Millspaugh, Brundige, Gitzen, & Raedeke, 2004; Vander Wal, Laforge, & McLoughlin, 2014), the individual variation in our analysis may be conservative when compared to other species of wildlife residing in other systems. In this way, the results of our analysis are consistent with an array of recent research demonstrating profound individuality in animal‐habitat relationships for a number of wildlife species (Bonnot et al., 2015; Réale et al., 2010; Spiegel et al., 2017). Among elk research, for example, certain individuals (e.g., matriarch females) have been observed to dictate the directionality and pace of movement and, correspondingly, the selection of available resources (Millspaugh et al., 2004; Putman & Flueck, 2011). An increased understanding of the role of individual variation will help to create more resolute animal‐habitat models which can account for tendencies of different members of the study population (Sawyer, Nielsen, Lindzey, & McDonald, 2006; Sawyer et al., 2007).

The development of individual‐level RSFs enabled us to explore the individuality of elk‐habitat relationships (Buskirk & Millspaugh, 2006; Marzluff et al., 2004; Millspaugh et al., 2006). Via this process, we discovered important variation in elk resource selection that would have otherwise been obscured via the process of aggregating animal locational data (Figure 1). For instance, we found that the number and composition of parameters featured in the most supported models varied across individual elk. The minimum number of parameters featured in the most supported model was seven, the maximum was seventeen, and the average was 14.4 parameters per elk. This variability in parameterization was masked in the population‐level model where the most parameterized model (the global model) was the most supported.

We also found that parameter estimates at the individual level were highly variable and often not commensurate with the variation identified by the *σ*_*k*_ estimates (representing variation among animal IDs as a random effect) of the population‐level model (Figure 2). Recent research has shown that random effects representing animal IDs may not be capable of sufficiently accounting for the individuality of animal‐habitat relationships (Pape & Löffler, 2015; Sawyer et al., 2006, 2007). That being said, we did detect a few parameters which had consistently similar estimates at the individual and population levels. For instance, the dispersion of parameter point estimates for slope at the individual level was well captured by the population‐level estimates (Figure 2a). Likewise, point estimates for interspersion index at the individual level mirrored estimates at the population level (Figure 2a). However, many other parameters had individual point estimates that greatly exceeded the bounds of the population‐level estimates (Figure 2b).

Furthermore, the maps representing the predicted relative probability of use for elk illustrated broad‐scale differences in selection strategies between individual‐ and population‐level models (Table 1; Figure 3). The low levels of correlation between individual‐ and population‐level predictions identified that almost half of the elk had patterns of habitat use that differed markedly from the predicted use at the population level (Table 1). These differences were particularly apparent in the northwest and southeast portions of the study area (Figure 3). This type of variation between individual‐level and population‐level modeling approaches is troubling, given that RSF maps are often developed to inform management and conservation action (Boyce, Vernier, Nielsen, & Schmiegelow, 2002; Hebblewhite & Merrill, 2008; Johnson, Seip, & Boyce, 2004). Maps like these are used to identify key habitats for species of conservation concern, to protect certain habitats so as to facilitate wildlife population goals, and to spatially delineate the location of human activities so as to minimize sources of anthropogenic disturbance on wildlife (Millspaugh, Rittenhouse, Montgomery, Matthews, & Slotow, 2015; Montgomery, Roloff, Millspaugh, & Nylen‐Nemetchek, 2014; Petrunenko et al., 2016). Thus, failure to develop accurate maps because of artifacts of the data collection or analytical process could prove problematic for meeting wildlife management objectives that tend to be developed at the population level.

The individual variation that we observed in this study was evident across all three of the metrics that we evaluated. However, in light of these results, we do not recommend that researchers abandon population‐level RSF modeling altogether. Similarly, we are not suggesting that conservation and management practice built upon population‐level RSFs is inherently flawed. Instead, we highlight that RSF modeling to date has been very useful and is in need of continued modification so as to maintain that utility moving forward. Specifically, we recommend that researchers and managers make efforts to model animal‐habitat relationships at the individual level and compare the output to the more conventional population‐level models. This exercise will be important to fully appreciate the consequences of variation among individuals on ecology, conservation, and management. We do recognize that running models at the individual level presents computational challenges. Development of computational shortcuts for estimation of hierarchical RSFs, however, is an area of active research (e.g., Hooten et al., 2016). Further, with the increasing availability of HPC resources and improvements to animal tracking technologies, data‐intensive computing is becoming more accessible (Kays et al., 2015).

The methods that we used here provide a framework that ecologists can follow to assess the individuality of animal‐habitat relationships in their study system. These approaches can be useful for evaluating the utility of population‐level inference from RSFs, which should be expected to vary among and across ecosystems and studies (Hanks, Hooten, Johnson, & Sterling, 2011). For this study, we accessed HPC services (Michigan State University Institute for Cyber‐Enabled Research) that greatly facilitated the handling of extensive amounts of locational data, the fitting of Bayesian RSFs, and the batch‐processing of large numbers of individual‐level models. Thus, we encourage researchers and managers to seek out collaborative partnerships with statisticians, biometricians, and HPC services to examine the consequences of individual variation in their own systems. In the end, we suspect that both individual‐ and population‐level models will be developed to generate valuable information informing ecology, evolution, and conservation.

## DATA ACCESSIBILITY

The data deriving from this research have been archived in a publicly available data repository Movebank (http://movebank.org) under the study name *Elk* (*C. elaphus*) *movement ecology in Missouri*.

## AUTHOR CONTRIBUTIONS

R.A. Montgomery and K. Redilla were the project leaders for this analysis and developed and executed every aspect of the assessment. T. Smith and B. Keller assisted with data collection and analysis. W. Ortiz and J.J. Millspaugh assisted with the generation of the question, data collection, methodological development, and the write‐up of the manuscript.
